# ASO Author Reflections: The Clinical Relevance of Sentinel Nodes in Minor Lymph Node Fields Such as the Triangular Intermuscular Space in Patients with Melanoma

**DOI:** 10.1245/s10434-023-13443-1

**Published:** 2023-05-03

**Authors:** Trine Schoenfeldt, Annette H. Chakera, Omgo E. Nieweg, John F. Thompson

**Affiliations:** 1 Melanoma Institute Australia, The University of Sydney, Sydney, NSW Australia; 2 Department of Plastic Surgery, Breast Surgery and Burns, Copenhagen University Hospital - Rigshospitalet, Copenhagen, Denmark; 3 Department of Clinical Medicine, The University of Copenhagen, Copenhagen, Denmark; 4 Department of Plastic Surgery, Copenhagen University Hospital–Herlev and Gentofte, Copenhagen, Denmark; 5 Faculty of Medicine and Health, The University of Sydney, Sydney, NSW Australia; 6 Department of Melanoma and Surgical Oncology, Royal Prince Alfred Hospital, Sydney, NSW Australia

## Past

For patients with melanoma, the frequency and clinical relevance of metastasis to sentinel lymph nodes (SLNs) in minor lymph node fields is controversial. The triangular intermuscular space (TIS) on the upper lateral back contains one such minor node field and is often overlooked.^1^ Usually containing one to three lymph nodes, it is bounded by teres major, teres minor and the long head of triceps, and efferent lymphatics pass anteriorly from nodes in the TIS into the posterior axilla. Unless lateral lymphoscintigram views are obtained, preferably with SPECT-CT also,^2^ nodes in the TIS are likely to be misidentified as axillary nodes. This probably explains why the largest hitherto reported series of patients with TIS SLNs contained only 14 patients.^3^

## Present

We assessed the frequency and management of SLNs in the TIS and outcomes following biopsy of these SLNs using information from a large institutional database.^4^ Preoperative lymphoscintigraphy revealed SLNs in the TIS of 266 patients, constituting 14% of 2,296 patients with melanomas on the upper back. SLN biopsy was performed in 53% of these patients, and 12% were found to be SLN-positive, resulting in upstaging of the majority of them. Recurrence in the TIS occurred only in patients who had not had a SLN biopsy, indicating that relapse in the TIS can be prevented by a SNB.

## Future

Our study demonstrated the importance of high-quality, preoperative lymphoscintigraphy with lateral views, preferably with SPECT/CT, for patients with melanomas on the upper back. Biopsy of SLNs identified in the TIS is necessary to ensure accurate staging, and without this, eligible patients may not be offered potentially effective, adjuvant, systemic therapy.^5^ The SNB procedure will also result in better regional disease control and improved disease-free survival. The same principles are likely to apply to SNBs in other minor lymph node fields in patients with melanoma.
